# Relating the gut metagenome and metatranscriptome to immunotherapy responses in melanoma patients

**DOI:** 10.1186/s13073-019-0672-4

**Published:** 2019-10-09

**Authors:** Brandilyn A. Peters, Melissa Wilson, Una Moran, Anna Pavlick, Allison Izsak, Todd Wechter, Jeffrey S. Weber, Iman Osman, Jiyoung Ahn

**Affiliations:** 1 Department of Population Health, NYU School of Medicine, New York, NY 10016 USA; 2 Department of Medicine, NYU School of Medicine, New York, NY USA; 3 NYU Perlmutter Cancer Center, New York, NY USA; 4 Present Address: Sidney Kimmel Cancer Center, Thomas Jefferson University, Philadelphia, PA USA; 5 The Ronald O. Perelman Department of Dermatology, NYU School of Medicine, New York, NY USA

**Keywords:** Melanoma, Immunotherapy, Microbiome, Metagenome, Metatranscriptome

## Abstract

**Background:**

Recent evidence suggests that immunotherapy efficacy in melanoma is modulated by gut microbiota. Few studies have examined this phenomenon in humans, and none have incorporated metatranscriptomics, important for determining expression of metagenomic functions in the microbial community.

**Methods:**

In melanoma patients undergoing immunotherapy, gut microbiome was characterized in pre-treatment stool using 16S rRNA gene and shotgun metagenome sequencing (*n* = 27). Transcriptional expression of metagenomic pathways was confirmed with metatranscriptome sequencing in a subset of 17. We examined associations of taxa and metagenomic pathways with progression-free survival (PFS) using 500 × 10-fold cross-validated elastic-net penalized Cox regression.

**Results:**

Higher microbial community richness was associated with longer PFS in 16S and shotgun data (*p* < 0.05). Clustering based on overall microbiome composition divided patients into three groups with differing PFS; the low-risk group had 99% lower risk of progression than the high-risk group at any time during follow-up (*p* = 0.002). Among the species selected in regression, abundance of *Bacteroides ovatus*, *Bacteroides dorei*, *Bacteroides massiliensis*, *Ruminococcus gnavus*, and *Blautia producta* were related to shorter PFS, and *Faecalibacterium prausnitzii*, *Coprococcus eutactus*, *Prevotella stercorea*, *Streptococcus sanguinis*, *Streptococcus anginosus*, and *Lachnospiraceae bacterium 3 1 46FAA* to longer PFS. Metagenomic functions related to PFS that had correlated metatranscriptomic expression included risk-associated pathways of l-rhamnose degradation, guanosine nucleotide biosynthesis, and B vitamin biosynthesis.

**Conclusions:**

This work adds to the growing evidence that gut microbiota are related to immunotherapy outcomes, and identifies, for the first time, transcriptionally expressed metagenomic pathways related to PFS. Further research is warranted on microbial therapeutic targets to improve immunotherapy outcomes.

## Background

Treatment with immunotherapy targeting checkpoint inhibitors PD-1 or CTLA-4 significantly increases survival in patients with metastatic melanoma over other standards of care [1, 2], with anti-PD-1 and anti-PD-1/CTLA-4 combination therapy emerging as most effective [3, 4]. However, responses to therapy are heterogeneous and not durable in large patient subsets: 3-year overall survival rates were 58%, 52%, and 34% in combination therapy, anti-PD-1, and anti-CTLA-4 groups, respectively [4]. Consequently, identification of host and tumor factors modulating treatment response is an area of active research to improve survival rates [5].

Recent evidence suggests that immunotherapy efficacy may be impacted by the gut microbiota, which profoundly shape the human immune system [6] and thus may play a role in antitumor T cell responses. In mice receiving anti-CTLA-4 immunotherapy, antitumor immunity was dependent on the presence of specific *Bacteroides* species [7]. Likewise, *Bifidobacterium* enhanced the efficacy of anti-PD-L1 immunotherapy in mice with melanoma [8]. In human melanoma patients undergoing immunotherapy, gut microbiome composition has been significantly associated with clinical response [9–12], and antitumor immunity was enhanced in germ-free mice receiving fecal transfer from the responding patients [9, 10]. However, results between studies thus far have been inconsistent regarding which species and metagenomic functions are related to immunotherapy response. Notably, most published studies have dichotomized patients into responders and non-responders, a practice which ignores time-to-event data and could result in loss of precision [13]. Additionally, published studies have not incorporated metatranscriptomic data into their analysis, which is crucial for understanding actual expression levels of metagenomic functions in the microbial community. Here, we robustly characterized the pre-immunotherapy gut microbiome in a pilot study of melanoma patients using 16S rRNA gene sequencing, shotgun metagenome sequencing, and shotgun metatranscriptome sequencing. We tested whether gut microbiome overall diversity and composition were related to progression-free survival using Cox proportional hazards models, and identified specific microbial taxa and functional pathways that were consistently related to progression-free survival in repeated cross-validation analyses.

## Methods

### Patients

Patients (*n* = 27) with metastatic melanoma scheduled to receive immunotherapy at NYU Langone Health were recruited into this study from September 2016 to November 2017. Follow-up for the current analysis occurred through September 2018. All patients pursuing treatment with immunotherapy were eligible for the study. The study was discussed with patients prior to starting treatment with immunotherapy, and all patients provided informed consent. Patients were enrolled into an IRB-approved institutional database and sample collection study (IRB#10362) and had prospective-driven follow-up. In addition, patients were seen routinely for treatment, and follow-up was performed at those visits as well. Stool kits were provided prior to starting treatment so as to obtain a pre-treatment stool sample. All demographic and clinical/pathological patient information was abstracted from electronic health records.

### Samples

Patients collected stool with provided kits at home prior to the start of immunotherapy. Kits included a stool collection tube with 10 ml RNAlater, instructions for stool collection, and a return addressed box with pre-paid postage. Patients were instructed to mail samples back within 1 day; upon receipt, samples were stored at − 80 °C until use.

### Definitions

The primary endpoint was progression-free survival (PFS), which included disease progression or death from any cause as events. Person time is defined as time from immunotherapy start date to event (first progression or death) or loss to follow-up (censored). Covariates included in statistical models (i.e., age, number of sites of metastases, stage, BMI) were based on electronic medical chart information prior to the start of immunotherapy rather than initial diagnosis, to coincide best with time of stool sample collection.

### 16S rRNA gene sequencing

#### Assay

Stool samples underwent 16S rRNA gene sequencing at the Environmental Sample Preparation and Sequencing Facility at Argonne National Laboratory, as previously described [14]. DNA was extracted using the Mo Bio PowerSoil DNA isolation kit, following the manufacturer’s protocol. The V4 region of the 16S rRNA gene was PCR amplified with the 515F/806R primer pair, which included sequencer adapter sequences used in the Illumina flow cell and sample-specific barcodes [15, 16]. Each 25 μL PCR reaction contained 9.5 μL of Mo Bio PCR Water (Certified DNA-Free), 12.5 μL of QuantaBio’s AccuStart II PCR ToughMix (2× concentration, 1× final), 1 μL Golay barcode tagged Forward Primer (5 μM concentration, 200 pM final), 1 μL Reverse Primer (5 μM concentration, 200 pM final), and 1 μL of template DNA. The conditions for PCR were as follows: 94 °C for 3 min to denature the DNA, with 35 cycles at 94 °C for 45 s, 50 °C for 60 s, and 72 °C for 90 s, with a final extension of 10 min at 72 °C. PCR products were quantified using PicoGreen (Invitrogen) and a plate reader (Infinite 200 PRO, Tecan). Sample PCR products were then pooled in equimolar amounts, purified using AMPure XP Beads (Beckman Coulter), and then quantified using a fluorometer (Qubit, Invitrogen). Molarity was then diluted to 2 nM, denatured, and then diluted to a final concentration of 6.75 pM with a 10% PhiX spike for sequencing on the Illumina MiSeq. Amplicons were sequenced on a 151 bp × 12 bp × 151 bp MiSeq run [16].

#### Sequence read processing

Sequence reads were processed using QIIME 2 [17]. Briefly, sequence reads were demultiplexed and paired-end reads were joined, followed by quality filtering as described in Bokulich et al. [18]. Next, the Deblur workflow was applied, which uses sequence error profiles to obtain putative error-free sequences, referred to as “sub”-operational taxonomic units (s-OTU) [19]. s-OTUs were assigned taxonomy using a naïve Bayes classifier pre-trained on the Greengenes [20] 13_8 99% OTUs, where the sequences have been trimmed to only include 250 bases from the 16S V4 region, bound by the 515F/806R primer pair. A phylogenetic tree was constructed via sequence alignment with MAFFT [21], filtering the alignment and applying FastTree [22] to generate the tree.

### Shotgun metagenome sequencing

#### Assay

Stool samples underwent shotgun metagenome sequencing at the Environmental Sample Preparation and Sequencing Facility at Argonne National Laboratory. DNA was extracted as above and quantified using a fluorometer (Qubit, Invitrogen). DNA was then mechanically sheared to the desired insert size of the final library using the Covaris S-series system, and products brought to 15 μL using Agencourt AMPure XP beads (Beckman Coulter). The Apollo 324 system (Takara Bio) was then used for end-repair, A-tailing, Illumina adaptor and barcode ligation, and size selection to generate the libraries. Libraries are run through 10–15 cycles of PCR with Kapa Biosystems Library Amplification kits, followed by further size selection with Blue Pippin Prep (Sage Science). Final library quantification is achieved using the Qubit Fluorometer (for concentration) and the Agilent 2100 Bioanalyzer (for library insert size and length). Libraries were sequenced on the Illumina HiSeq 2500 on a 2 × 101 bp paired-end run.

#### Sequence read processing

Reads were demultiplexed, and Trimmomatic [23] was used for read length filtering, trimming of Illumina adapter sequences, and trimming of low-quality read ends. Reads mapping to the human genome were identified using Bowtie2 [24] and removed. Forward and reverse reads were concatenated for input into the taxonomic and functional profiling tools, MetaPhlAn2 and HUMAnN2. MetaPhlAn2 [25] uses a set of ~ 1 million clade-specific markers (average 184 marker genes for each species) from > 7500 species to unequivocally identify and quantify specific microbial clades at the species level or higher. HUMAnN2 maps reads to functionally annotated microbial species genomes and uses a translated search to align unmapped reads to UniRef90 protein clusters [26] (gene families). Gene families are then grouped into MetaCyc pathways [27] using MinPath [28]. For a lower level of resolution, we also regrouped UniRef90 gene families into MetaCyc reactions using the “humann2_regroup_table” script. We removed unintegrated/unmapped/unknown/ungrouped pathways, reactions, and gene families prior to calculating relative abundance, using the “humann2_renorm_table” script.

### Shotgun metatranscriptome sequencing

#### Assay

Stool samples underwent shotgun metatranscriptome sequencing at the Environmental Sample Preparation and Sequencing Facility at Argonne National Laboratory. RNA was extracted using the Mo Bio PowerMicrobiome RNA Isolation Kit and quantified using a Qubit Fluorometer. RNA integrity and size distribution were determined using the Agilent RNA 6000 Nano Kit on the Agilent 2100 Bioanalyzer. Samples then underwent DNase treatment using the Turbo DNA-free kit (Life Technologies), and ribosomal depletion using the Ribo-Zero rRNA Removal Kit (Bacteria) (Illumina). Bacterial mRNA purification was achieved with AMPure RNAClean XP Beads, and cDNA libraries were generated using the ScriptSeq V2 RNA-Seq Library Preparation Kit (Illumina). Libraries were sequenced on the Illumina HiSeq 2500 on a 2 × 151 bp paired-end run. In this study, metatranscriptomic library preparation failed for 10 samples due to poor RNA quality; thus, only a subset of 17 patient samples underwent metatranscriptomic sequencing.

#### Sequence read processing

Reads were processed in the same way as the shotgun metagenome samples, with the exception of removing reads with Bowtie2 mapping to the human transcriptome, rather than human genome. Paired metagenomic taxonomic profiles were used as taxonomic profile inputs for HUMANnN2. In addition to relative abundance of gene families, reactions, and functional pathways metatranscriptomic expression, we also derived relative expression (i.e., independent of gene copy number) using the “humann2_rna_dna_norm” script on these three levels of data.

### Statistical analysis

#### α-Diversity

α-diversity (within-sample microbiome diversity) was assessed based on the 16S rRNA gene and shotgun sequencing data, using richness (number of s-OTUs [16S] or subspecies [shotgun]) and the Shannon diversity index. For 16S, these indices were calculated in 100 iterations of s-OTU tables rarefied to 18,368 sequence reads per sample, which was the lowest sequencing depth among samples, using the QIIME 2 diversity plugin. The final value for each sample was calculated by averaging over the 100 iterations. For shotgun, these indices were calculated on the subspecies-level data without rarefaction. The subspecies level of the shotgun data includes both strains and species (for species with no strain classification). We used Cox proportional hazards models to determine whether α-diversity was associated with progression-free survival, adjusting for age, sex, BMI, stage, number of sites of metastases, and antibiotic use in the last 6 months.

#### β-Diversity

β-diversity (between-sample microbiome diversity) was assessed based on the 16S rRNA gene and shotgun sequencing data using the weighted UniFrac distance [29] (16S only) and the Jensen-Shannon Divergence (JSD) [30]. Distances were calculated on the s-OTU (16S) or subspecies (shotgun) level. Principal coordinate analysis (PCoA) [31] was used for visualization. The community-level test of association between the microbiota and survival times (MiRKAT-S) [32] and the optimal microbiome-based survival analysis test (OMiSA) [33] were used to test the association of overall bacterial composition with progression-free survival, adjusting for age, sex, BMI, stage, number of sites of metastases, and antibiotic use in the last 6 months. We also assigned samples to clusters by applying Ward’s Hierarchical Agglomerative Clustering method [34] to the distance matrices, and then tested whether these clusters were related progression-free survival using log-rank tests.

#### Identification of taxa

Genera, species, and subspecies (or sub-OTUs) associated with progression-free survival were assessed independently in the 16S and shotgun metagenome datasets using repeated cross-validated elastic-net penalized Cox proportional hazards regression. 16S s-OTUs were agglomerated into genus and species levels; this was not necessary for MetaPhlAn2 (shotgun) output which is already in a taxonomic level format. Taxonomic abundance was transformed using the centered log ratio (clr) transformation [35, 36] after adding a pseudocount, in order to remove compositional constraints of sequencing. 16S agglomerated genera or species missing genus- or species-level classification, respectively, were removed for this analysis. Likewise, shotgun taxa missing classification at the genus, species, or subspecies levels were removed from the respective levels. Additionally, we only tested taxa present in at least 25% of samples and with mean relative abundance greater than 0.01% in order to minimize the number of tests. These exclusions resulted in inclusion of 42 16S genera, 24 16S species, and 233 16S s-OTUs, and 43 shotgun genera, 110 shotgun species, and 65 shotgun subspecies for testing. We conducted 500 × 10-fold cross-validated elastic-net penalized Cox regression using the “cv.glmnet” function in the glmnet R package [37], with an *α* value of 0.5 to allow groups of correlated predictors to be selected together. Non-penalized covariates (age, sex, BMI, stage, number of sites of metastases, and antibiotic use in the last 6 months) were included in each model. We summed the number of times each taxon was selected out of the 500 repetitions. For all tested taxa, we also fit standard Cox proportional hazards models for progression-free survival, adjusting for the covariates listed above. *p* values for these models were adjusted for the false discovery rate (FDR) [38]; FDR adjustment was done at each taxonomic level (i.e., genus, species) separately. We focused further on taxa selected ≥ 25% of the 500 times (125 times or more) and with FDR-adjusted *q* < 0.20 in either the 16S or shotgun data; for these, we compared hazard ratios and examined correlations between the two data types to confirm findings and confirm taxonomic identities.

#### Identification of gene families, reactions, and functional pathways

We assessed associations of metagenomic functional pathways, reactions, and gene families’ relative abundance with progression-free survival. Pathways, reactions, and gene families were transformed using the centered log ratio (clr) transformation [35, 36] after adding a pseudocount, in order to remove compositional constraints of sequencing. We only assessed pathways/reactions/gene families present in at least 25% of samples, with mean relative abundance greater than 0.01% (for gene families) or 0.03% (for pathways/reactions) and, among these, with variance greater than the 25th percentile of variances, in order to minimize the number of tests that are unlikely to result in significant findings. This resulted in inclusion of 177 metagenomic pathways, 662 reactions, and 146 gene families. 500 × 10-fold cross-validated elastic-net penalized Cox regression, as described above in “Identification of taxa”, was used to identify functional pathways, reactions, and gene families related to progression-free survival. We used Spearman’s correlation to examine associations between relative abundance of metagenomic features and their corresponding relative abundance in the metatranscriptome, and focused on metagenomic features with (a) selection ≥ 25% of the 500 times, (b) FDR-adjusted *q* < 0.20, and (c) correlated metatranscriptomic expression (*p* < 0.05). Using the same procedure, we also examined metatranscriptomic expression and relative expression of pathways/reactions/gene families related to progression-free survival; we considered this analysis exploratory due to a reduced sample size (*n* = 17).

## Results

Among the patients included in the current study, 12 progressed over the course of follow-up, which ranged from 10 to 25 months. The majority of the patients were male (78%) and white (96%), and 41% of the patients were receiving adjuvant immunotherapy (i.e., complete resection prior to therapy) (Table 1). Patients who progressed tended to be older and have lower BMI at baseline than patients who remained progression free (Table 1).

**Table 1:** Demographic and clinical characteristics of melanoma patients on immunotherapy

Characteristic	All patients (*n* = 27)	No progression (*n* = 15)	Any progression (*n* = 12)	*p* ^a^
Age (years)^b^, mean ± SD	70.3 ± 11.9	66.6 ± 12.5	74.9 ± 9.6	< 0.0001
Male, %	77.8	73.3	83.3	0.66
White, %	96.3	100	91.7	0.44
BMI (kg/m^2^)^b^, mean ± SD	27.5 ± 4.8	28.4 ± 4.3	26.5 ± 5.3	< 0.0001
Melanoma type, %				0.02
Nodular	18.5	6.7	33.3	
Acral lentiginous	3.7	0	8.3	
Superficial spreading	3.7	0	8.3	
Desmoplastic	3.7	0	8.3	
NOS/missing	70.4	93.3	41.7	
Driver mutation, %				0.83
NRAS	18.5	13.3	25	
BRAF	25.9	33.3	16.7	
None	29.6	26.7	33.3	
Missing	25.9	26.7	25	
Stage^b^, %				0.68
III	33.3	40	25	
IV	66.7	60	75	
LDH > 618 U/L^b^, %	7.4	0	16.7	0.22
Sites of metastasis^b,c^, %				0.21
0	40.7	53.3	25	
1–2	33.3	33.3	33.3	
≥ 3	25.9	13.3	41.7	
Immunotherapy type, %				0.44
Anti-PD-1	51.9	46.7	58.3	
Anti-CTLA-4	3.7	0	8.3	
Anti-PD-1/anti-CTLA-4	44.4	53.3	33.3	
Antibiotics in prior 6 months, %	55.6	60	50	0.71

^a^*p* value for difference by progression status, from Wilcoxon rank-sum test for continuous variables or Fisher’s exact test for categorical variables

^b^Characteristic prior to immunotherapy start (not at diagnosis)

^c^Patients with 0 sites of metastasis were resected with no evidence of disease and were being treated adjuvantly

Higher microbiome community richness was associated with longer progression-free survival (number of 16S s-OTUs: HR [95% CI] = 0.97 [0.95, 1.00], *p* = 0.02; number of shotgun subspecies: HR [95% CI] = 0.89 [0.79, 0.99], *p* = 0.03), adjusting for the covariates of age, sex, BMI, stage, number of sites of metastases, and antibiotic use in the last 6 months. Higher community diversity, as measured by the Shannon index, was associated with longer progression-free survival in the 16S data (*p* = 0.02) but not in the shotgun data (*p* = 0.90) (Additional file 1: Table S1). Results were similar with additional adjustment for immunotherapy regimen (monotherapy or combined therapy) (Additional file 1: Table S1).

**Fig. 1:**
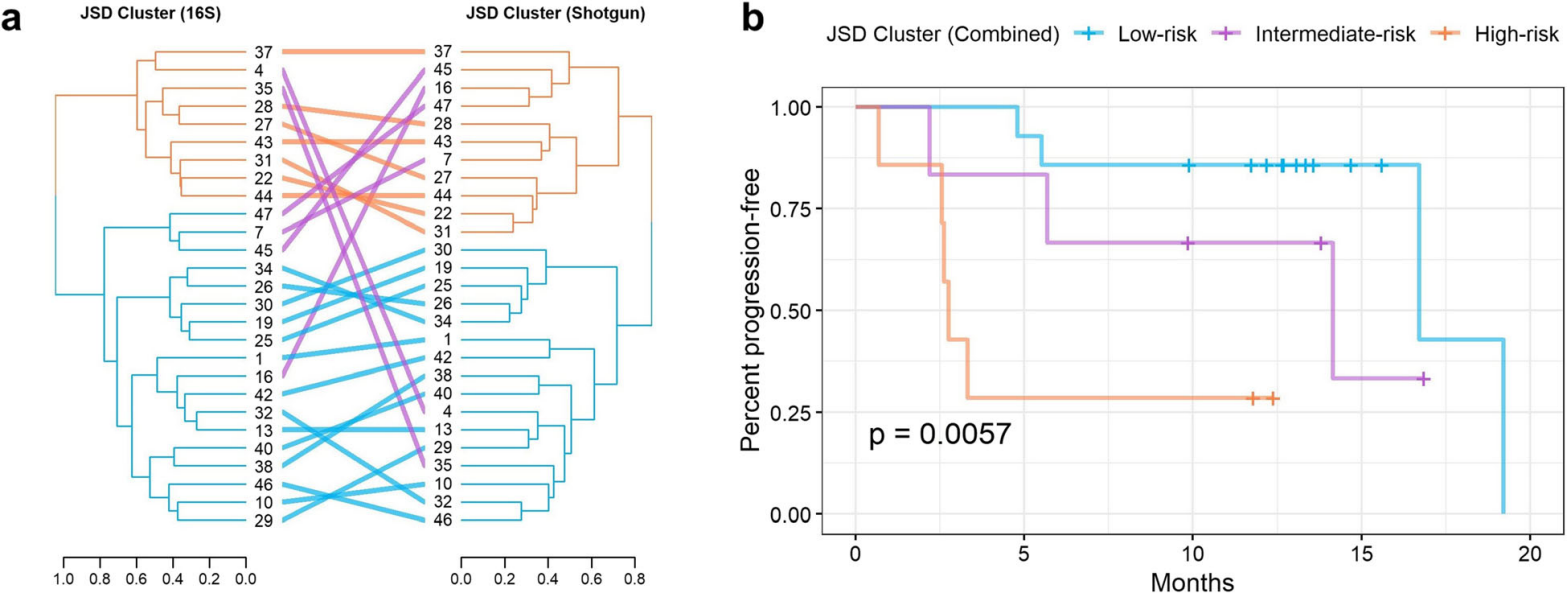
Patient clusters based on overall microbiome composition in 16S and shotgun data are related to progression-free survival. Ward’s Hierarchical Agglomerative Clustering method was used on the Jensen-Shannon Divergence (JSD) from the 16S s-OTU data and shotgun subspecies data to cluster patients into groups. **a** The dendrograms from 16S and shotgun were compared, and patients were assigned to two concordant groups (orange and blue) or a discordant group (purple). **b** The Kaplan-Meier curves of the patient groupings had significantly different progression-free survival (log-rank *p* = 0.0057)

In both the 16S and shotgun data, hierarchical clustering based on the JSD clustered patients into two groups, and these two groups significantly differed in their progression-free survival (16S log-rank *p* = 0.005; shotgun log-rank *p* = 0.02) (Additional file 2: Figure S1). We then further grouped patients as follows: a “low-risk” group comprised of patients concordantly in the low-risk 16S and low-risk shotgun clusters; a “high-risk” group comprised of patients concordantly in the high-risk 16S and high-risk shotgun clusters; and an “intermediate-risk” group comprised of patients discordant between the 16S and shotgun clusters (Fig. 1a). These groups differed significantly in their progression-free survival (log-rank *p* = 0.006; Fig. 1b). Additionally, these groups were related to progression-free survival in Cox proportional hazards models adjusting for covariates: patients in the low-risk group had 99% lower risk of progression than the high-risk group at any time during follow-up (HR [95% CI] = 0.01 [0.001, 0.20], *p* = 0.002), while patients in the intermediate-risk group had non-significantly lower risk (HR [95% CI] = 0.10 [0.005, 2.17], *p* = 0.14) (Additional file 1: Table S1).

In the MiRKAT-S test, overall microbiome community composition as measured by the JSD was marginally related to progression-free survival for both the 16S (*p* = 0.09) and shotgun (*p* = 0.06) data, adjusting for covariates (Additional file 1: Table S1). Measures relying on the phylogenetic tree (weighted UniFrac, OMiSA) could only be assessed in the 16S data; community composition as measured by the weighted UniFrac was marginally associated with progression-free survival (*p* = 0.07), while the OMiSA test was not significant (*p* = 0.17) (Additional file 1: Table S1).

**Fig. 2:**
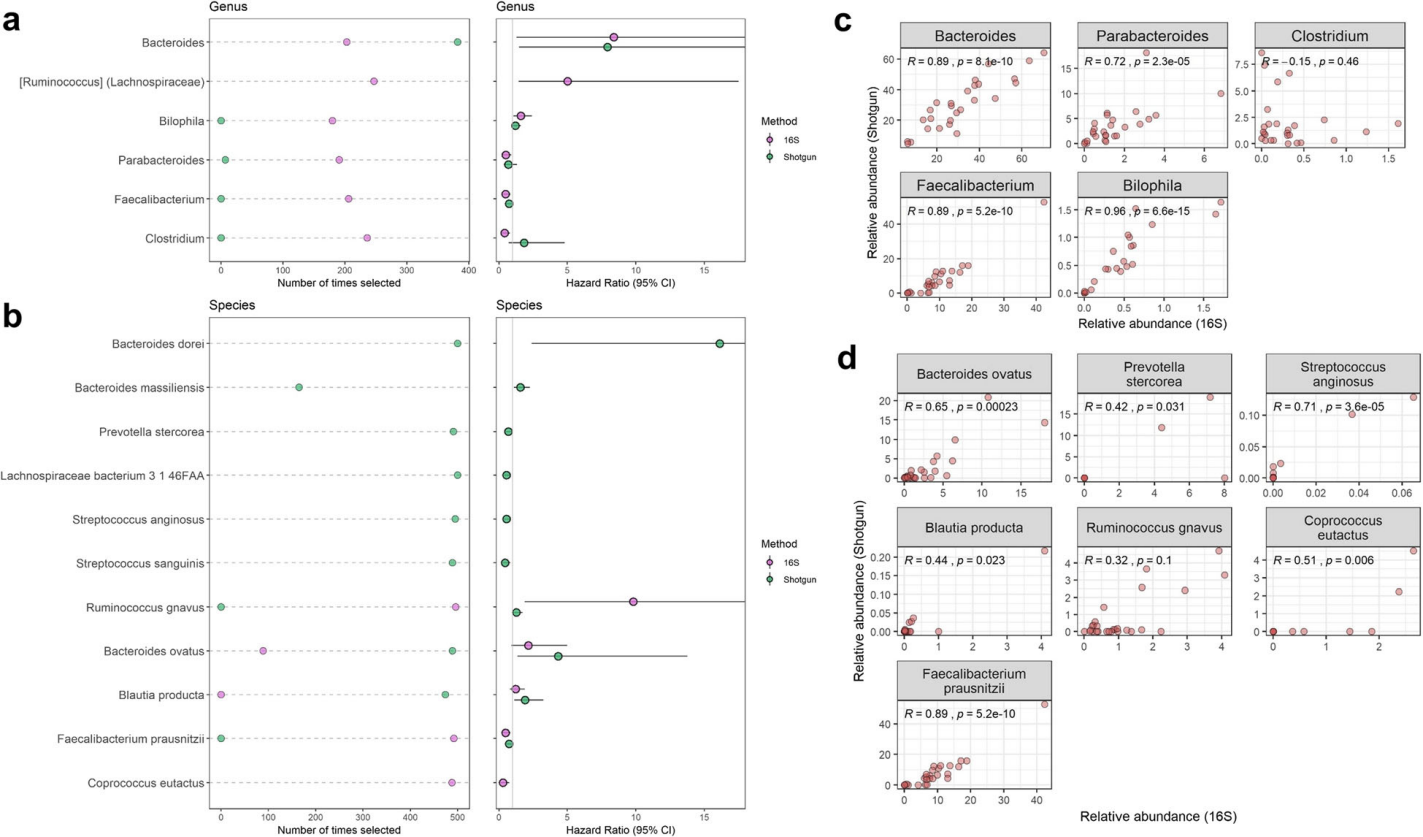
Genera and species related to progression-free survival. **a**, **b** For genera or species selected > 125 times in 500 × 10-fold cross-validated elastic-net penalized Cox regression and with FDR-adjusted *q* < 0.20 in either the 16S or shotgun data, we show number of times selected and the hazard ratio. Note: not all genera and species were detected in both the 16S and shotgun data. **c**, **d** Scatterplots of 16S vs. shotgun relative abundance of genera and species, for genera and species selected in the regression and detected in both the 16S and shotgun data. Spearman’s rho and *p* value are displayed on the plots

In 500 × 10-fold cross-validated elastic-net Cox regression models for progression-free survival adjusting for covariates, 6 genera and 3 species were selected > 25% of the time with *q* < 0.20 in the 16S data, and 8 species and 4 subspecies were selected > 25% of the time with *q* < 0.20 in the shotgun data (Fig. 2a, b; Additional file 1: Table S2; Additional file 2: Figure S2). There were no 16S s-OTUs or shotgun genera which met the cut-off criteria (Additional file 1: Table S2). Most of the genera and species selected in either the 16S or shotgun data that were present in both datasets were highly correlated between the two datasets (Fig. 2c, d) and showed consistent associations with progression-free survival in both datasets. These included genera *Bacteroides* and *Bilophila*, and species *Bacteroides ovatus*, *Blautia producta*, and *Ruminococcus gnavus*, associated with shorter progression-free survival, and genera *Faecalibacterium* and *Parabacteroides* and species *Faecalibacterium prausnitzii*, associated with longer progression-free survival. Genus *Clostridium* was not well correlated between the 16S and shotgun datasets and was only associated with longer progression-free survival in the 16S data, while *Coprococcus eutactus* was associated with longer progression-free survival in the 16S data but had insufficient abundance to be tested in the shotgun data (Fig. 2). As to be expected, many of the species selected in the shotgun data were not detected, either at all or with sufficient abundance, in the 16S data, including *Bacteroides dorei* and *Bacteroides massiliensis*, associated with shorter progression-free survival, and *Prevotella stercorea*, *Lachnospiraceae bacterium 3 1 46FAA*, *Streptococcus anginosus*, and *Streptococcus sanguinis*, associated with longer progression-free survival (Fig. 2b).

**Fig. 3:**
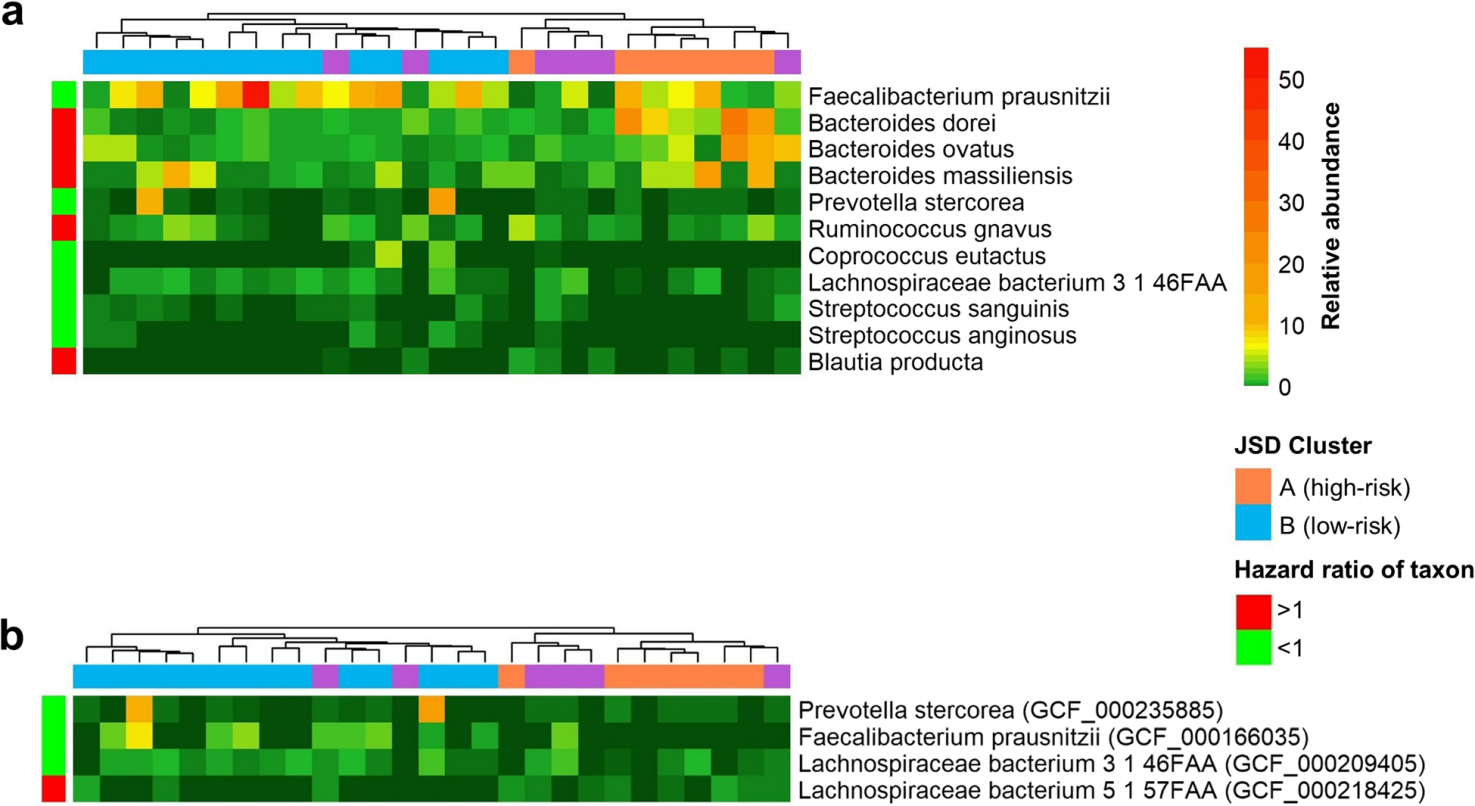
Heatmap of shotgun species and subspecies relative abundance. Relative abundance of **a** species and **b** subspecies in the shotgun data; only those selected > 125 times in 500 × 10-fold cross-validated elastic-net penalized Cox regression and with FDR-adjusted *q* < 0.20 in either the 16S or shotgun data are shown. Ward’s Hierarchical Agglomerative Clustering method was used, column (patient) distance was based on the shotgun JSD (from Fig. 1), and row (species) distance on the Manhattan distance. Species and subspecies are annotated with the direction of their hazard ratio with progression-free survival, and patients are annotated with their combined JSD cluster (from Fig. 1)

Relative abundance of the selected species and subspecies (based on shotgun data) tended to differ between the high-risk and low-risk JSD cluster groups (Fig. 3a, b). Taxa associated with shorter progression-free survival, such as species *Bacteroides ovatus*, *Bacteroides dorei*, *Bacteroides massiliensis*, and *Blautia producta*, and subspecies of *Lachnospiraceae bacterium 5 1 57FAA*, were elevated in the high-risk group, while taxa associated with longer progression-free survival, such as species *Streptococcus sanguinis* and *Streptococcus anginosus*, and subspecies of *Prevotella stercorea*, *Faecalibacterium prausnitzii*, and *Lachnospiraceae bacterium 3 1 46FAA*, were elevated in the low-risk group (Fig. 3a, b).

**Fig. 4:**
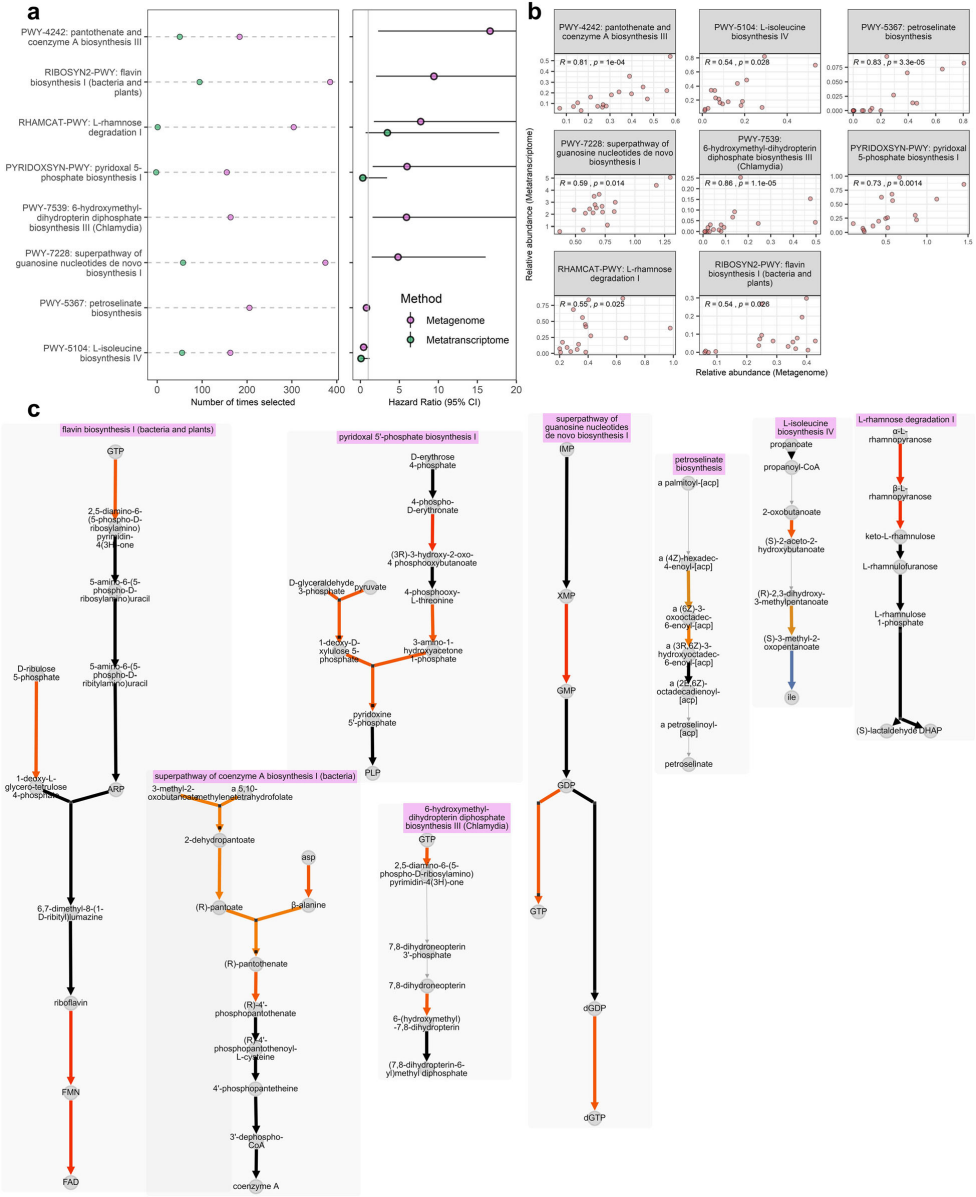
Metagenomic functional pathways related to progression-free survival. For metagenomic pathways selected > 125 times in 500 × 10-fold cross-validated elastic-net penalized Cox regression, that also had FDR-adjusted *q* < 0.20 and correlated (*p* < 0.05) metatranscriptomic expression, we show **a** number of times selected and the hazard ratio (alongside parallel data from the metatranscriptomic analysis in *n* = 17) and **b** correlations between metagenomic and metratranscriptomic functional pathway relative abundance. Spearman’s rho and *p* value are displayed on the plots. **c** MetaCyc pathway layouts for the pathways in (**a**, **b**). Each arrow represents one MetaCyc reaction, color-coded by its hazard ratio in the metagenomic analysis. Arrows in black represent reactions not tested due to low carriage, abundance, or variance of the reaction

In repeated cross-validated Cox regression for metagenomic functional pathways, reactions, and gene families, 11 pathways, 26 reactions, and 16 gene families were selected > 25% of the time with *q* < 0.20 (Additional file 1: Table S3-S5). Among these, 8 pathways, 17 reactions, and 7 gene families had significant positive correlations (*p* < 0.05) with metatranscriptomic expression (Fig. 4, Additional file 2: Figures S3-S4). Selected metagenomic pathways with correlated metatranscriptomic expression that were related to longer progression-free survival included biosynthesis pathways for l-isoleucine and petroselinate. Those related to shorter progression-free survival included biosynthesis pathways for 6-hydroxymethyl-dihydropterin diphosphate, pantothenate and coenzyme A, flavin, pyridoxal 5-phosphate, and guanosine nucleotides, and the degradation pathway for l-rhamnose (Fig. 4a, b; Additional file 1: Table S3). Within each of these pathways, abundance of specific biochemical reactions were also related to progression-free survival, typically in the same direction as the parent pathway (Fig. 4c). Selected metagenomic reactions with correlated metatranscriptomic expression that were related to shorter progression-free survival included reactions involved in nucleotide phosphorylation and biosynthesis, l-rhamnose degradation, pectin degradation, and aerobic respiration (Additional file 1: Table S4, S6; Additional file 2: Figure S3). Most of the selected gene families with correlated metatranscriptomic expression were uncharacterized proteins; results for these gene families are shown in Additional file 1: Table S5 and Additional file 2: Figure S4. In repeated cross-validated Cox regression for metatranscriptomic expression and relative expression of functional pathways, reactions, and gene families, we did not identify features meeting our selection criteria, likely due to the smaller sample size (*n* = 17) available for this analysis (Additional file 1: Table S3-S5).

**Fig. 5:**
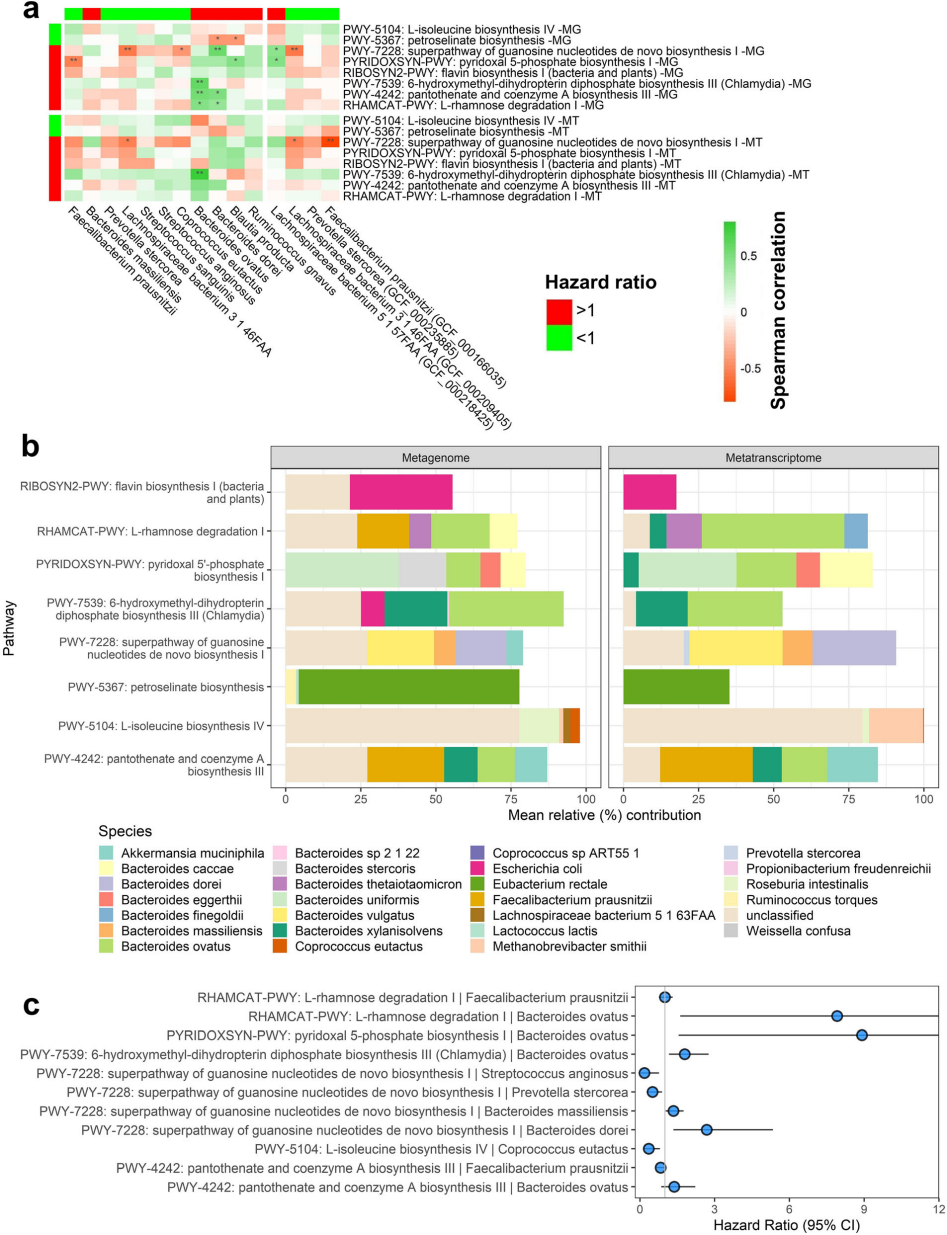
Contribution of shotgun metagenome taxa to shotgun metagenome and metatranscriptome functional pathways. **a** Spearman’s correlations are shown for shotgun species and subspecies vs. shotgun metagenome and metatranscriptome pathways. Only taxa selected in repeated cross-validated elastic-net penalized Cox regression are shown, and only pathways selected in regression and that had correlated metatranscriptomic expression are shown. Taxa and pathways relative abundance were used for correlation analysis. Taxa and pathways are annotated with the direction of their hazard ratio with progression-free survival in the metagenomic analysis. *p < 0.05; **p < 0.01. **b** Mean percent contribution of species to functional pathways in the metagenome and metatranscriptome data. Per-species pathway abundance values were normalized to 100% for each pathway within each patient individually, and means were taken across patients; here, we show the mean percent contribution for the top 5 contributing species to each pathway. **c** Hazard ratios for species-specific pathway abundances; all species-by-pathway combinations existing in the data (for our selected species and pathways) are shown

Risk-associated metagenomic pathways tended to be positively correlated with risk-associated species/subspecies and negatively correlated with protective species (Fig. 5a). For example, risk-associated *Bacteroides dorei* and *Bacteroides ovatus* were positively associated with l-rhamnose degradation and pantothenate and coenzyme A biosynthesis. Protective metagenomic pathways did not correlate strongly with protective species/subspecies. Similar correlation patterns were observed for species/subspecies with metatranscriptomic expression of these pathways (Fig. 5a). We next explored average species contributions to overall metagenome and metatranscriptome pathway abundances in this patient population (Fig. 5b); while multiple species are involved in each pathway, we noted that *Bacteroides ovatus* was a significant contributor to degradation of l-rhamnose and biosynthesis of pyridoxal 5-phosphate, 6-hydroxymethyl-dihydropterin diphosphate, and pantothenate and coenzyme A, while *Bacteroides dorei* was a significant contributor to guanosine nucleotides biosynthesis (Fig. 5b). Analysis of per-species pathway abundances with progression-free survival implied that degradation of l-rhamnose and biosynthesis of pyridoxal 5-phosphate, 6-hydroxymethyl-dihydropterin diphosphate, and pantothenate and coenzyme A by *Bacteroides ovatus* was related to shorter progression-free survival; that guanosine nucleotides biosynthesis by *Bacteroides dorei* and *Bacteroides massiliensis* was related to shorter progression-free survival; and that l-isoleucine biosynthesis by *Coprococcus eutactus* was related to longer progression-free survival (Fig. 5c).

## Discussion

In this pilot study of melanoma patients treated with immunotherapy, we observed a relationship between overall microbiome composition and risk of progression during follow-up. Clustering of patients based on the underlying microbial composition in their stool revealed patient groups with significantly different progression-free survival, including a high-risk group enriched in *Bacteroides* species, and a low-risk group enriched in *Faecalibacterium prausnitzii* and other protective species. Further, we observed metagenomic functions related to progression-free survival that had correlated metatranscriptomic expression and may serve as mechanisms for bacteria to influence immunotherapy response, including protective pathways of amino acid biosynthesis and risk-associated pathways of sugar degradation, guanosine nucleotide biosynthesis, and B vitamin biosynthesis. Finally, we observed that greater microbiome community richness was significantly associated with prolonged progression-free survival. Many of these results are consistent with previous literature, highlighting emergent bacterial modulators of immunotherapy treatment response.

While several studies now suggest that the gut microbiome is a critical player in immunotherapy response, the mechanisms by which this may occur remains unclear. The main principle of immunotherapy is to block immunosuppressive T cell checkpoints, allowing cytotoxic T cells to attack tumors [39]. Human gut microbiota may modulate the effectiveness of immunotherapy, and anticancer immunosurveillance in general, by shaping both effector and suppressor immune cell populations through pathogen-associated molecular patterns (PAMPs), antigens, and metabolites [40]. One hypothesis by which this occurs is via microbial proteins that mimic tumor antigens, resulting in T cell cross-reactivity; T cells may be primed by microbial antigens in the gut and travel to tumor sites, or the gut microbes or microbial antigens themselves may translocate to distant sites to induce a local T cell response near the tumor [40]. A second hypothesis is that gut microbes or microbial products activate pattern recognition receptors (PRRs), which stimulate the production of cytokines and interferons, thus leading to immunostimulatory or immunosuppressive reactions in T cells; PRR activation may occur in the gut and stimulate traveling innate immune cells, or the microbes or microbial products themselves may translocate [40]. Evidence for these hypothetical mechanisms underlines the potential causal impact of the gut microbiota on immunotherapy response and supports the future use of microbiome manipulation to increase the efficacy of immunotherapy [40].

To our knowledge, four studies have examined the relationship of the gut microbiome to immunotherapy response in human patients with melanoma. In 2018, Gopalakrishnan et al. reported higher α-diversity, higher relative abundance of *Faecalibacterium prausnitzii*, and lower relative abundance of Bacteroidales, in anti-PD-1 immunotherapy responders (*n* = 30) compared to non-responders (*n* = 13) [9]. They quantified T cell densities in pre-treatment tumors and peripheral blood and observed significant positive correlations of gut *Faecalibacterium* relative abundance with tumor CD8+ T cell infiltrate and peripheral CD8+ T cell and effector CD4+ T cell frequencies, while Bacteroidales was inversely related to these markers. Further, they found that gut *Faecalibacterium* was positively related to a peripheral cytokine profile favorable for response to anti-PD-1 immunotherapy, while Bacteroidales was related to a blunted peripheral cytokine response and to higher peripheral frequencies of immunosuppressive regulatory T cells and myeloid-derived suppressor cells [9]. Germ-free mice receiving fecal transplant from responding patients had reduced tumor size and enhanced antitumor T cell responses compared to mice receiving fecal transplant from non-responding patients [9]. Also in 2018, Matson et al. reported higher relative abundance of *Bifidobacterium longum*, *Collinsella aerofaciens*, and *Enterococcus faecium* and lower relative abundance of *Ruminococcus obeum* and *Roseburia intestinalis*, in anti-PD-1 immunotherapy responders (*n* = 16) compared to non-responders (*n* = 26) [10]. They too administered fecal material of responders and non-responders to germ-free mice via gavage, and found improved tumor control and enhanced T cell responses in the mice receiving fecal material from responding compared to non-responding patients [10]. In 2017, Frankel et al. reported enrichment of *Bacteroides caccae* in immunotherapy responders (*n* = 24) compared to non-responders (*n* = 15); among ipilimumab (anti-CTLA-4) + nivolumab (anti-PD-1) responders, they observed enrichment of *Faecalibacterium prausnitzii*, *Bacteroides thetaiotamicron*, and *Holdemania filiformis*, while in pembrolizumab (anti-PD-1) responders they observed enrichment of *Dorea formicigenerans* [11]. Finally, in another 2017 report, Chaput et al. observed higher relative abundance of *Faecalibacterium*, *Gemmiger*, and *Clostridium XIVa* and lower abundance of *Bacteroides*, in anti-CTLA-4 responders (*n* = 9) compared to non-responders (*n* = 17) [12]. Two of these studies have found that *Bacteroides* is related to poor immunotherapy response, while *Faecalibacterium* is related to improved response, consistent with our findings here. However, some of these previous studies have identified response-related taxa that were not significant in our study, and vice versa, we have identified response-related taxa that were not related to response in previous studies.

Inconsistent results between studies regarding the central species involved in immunotherapy response could be due to small sample sizes and differing populations under study. However, it is possible that the functional capacities of the microbiota (which can be redundant across species) are the more key determinant of immunotherapy responses rather than individual species. For this reason, we have characterized the metagenomes and metatranscriptomes of the study patients, to identify metagenomic functions expressed by the microbial community that may influence patient outcomes. We observed that a sugar degradation pathway (l-rhamnose), B vitamin biosynthesis pathways (pantothenate, pyridoxal 5-phospate, flavin, and 6-hydroxymethyl-dihydropterin diphosphate [folate precursor]), and guanosine nucleotide biosynthesis pathways were associated with shorter progression-free survival. Lactate, a product of sugar degradation, is known to drive tumor progression via its use by cancer cells as a nutrient source and by its promotion of tumor inflammation and inhibitory effect on cytotoxic T cells [41, 42]. It is not clear how microbial B vitamin biosynthesis may diminish immunotherapy response; yet interestingly, metagenomic pantothenate and riboflavin biosynthesis were both associated with resistance to colitis in melanoma patients on anti-CTLA-4 immunotherapy [43], perhaps indicative of their effects on immunity. We also observed that biosynthesis of the amino acid l-isoleucine was associated with longer progression-free survival. With mechanisms again unclear, Gopalakrishnan et al. also highlighted that metagenomic amino acid biosynthesis predominated in melanoma patients who responded to immunotherapy [9], though no specific amino acids were identified. Biosynthesis of the fatty acid petroselinate was also related to longer progression-free survival, though reactions within this pathway were not, making this a somewhat unstable finding. Finally, we observed that pectin degradation reactions were associated with shorter progression-free survival, leading us to infer that anticancer properties of pectin [44] may be disrupted by bacterial degradation.

Our study is strengthened by the robust assessment of the gut microbiome via 16S rRNA gene, shotgun metagenome, and for the first time, shotgun metatranscriptome sequencing. This allowed us to focus on expressed metagenomic functions potentially related to prognosis, which may be important for identifying adjuvant therapeutic targets for metagenomic functions, rather than specific species. We were additionally able to replicate our findings with the two primary flavors of microbiome profiling—targeted 16S amplicon sequencing and broad metagenomic shotgun sequencing. With analysis of 16S rRNA gene and shotgun metagenome data side by side, we were able to confirm the robustness of our findings with two data types. Clustering of patients based on 16S microbiome composition was slightly more predictive of progression-free survival than clusters based on shotgun microbiome composition, but species-level classification was much higher in the shotgun data, permitting us to identify more response-associated species than with 16S data alone. A further strength of our study was the long follow-up of patients and our use of survival analysis rather than dichotomization of patients into responders and non-responders, a practice done in previous studies [9–11] which may result in loss of precision [13].

Our study is limited by its small sample size, like other studies which came before; yet our replication of some findings from those previous studies, such as the relationship of *Faecalibacterium prausnitzii* and *Bacteroides* with immunotherapy outcomes, is encouraging. A further limitation of our study is that we had insufficient sample size to analyze adjuvant and metastatic patient groups separately and thus present a combined analysis of adjuvant and metastatic patients as one group. Though we did not observe differences in pre-treatment gut microbiome composition between adjuvant and metastatic patients (Additional file 2: Figure S6), we were unable to examine heterogeneity of microbiome effects on survival. Similarly, sample size was insufficient to analyze patient groups separately by immunotherapy treatment regimen; this type of analysis will be important to determine which immunotherapies could be enhanced most by an optimal microbiome composition. Finally, metatranscriptome sequencing data was only available for a subset of 17 patients, which limited our power to assess the relationship of metatranscriptomic expression and relative expression with progression-free survival.

## Conclusions

In conclusion, our pilot study results support the notion that the gut microbiota modulate response to immunotherapy in melanoma patients. Larger studies with robust microbiome characterization are needed to validate the microbial species and functions related to progression-free survival in melanoma patients on immunotherapy, and whether these relationships differ for adjuvant and metastatic patients or by immunotherapy type. Ultimately, this research may provide microbial therapeutic targets to improve immunotherapy outcomes and increase survival in these patients.

## Supplementary information

**Additional file 1.** Supplementary Tables S1-S6.

**Additional file 2.** Supplementary Figures S1-S6.

## Data Availability

The datasets supporting the conclusions of this article are available in the Sequence Read Archive repository, under BioProject accession number PRJNA541981 [45].

## Abbreviations

clr: Centered log ratio
HR: Hazard ratio
JSD: Jensen-Shannon Divergence
PAMP: Pathogen-associated molecular pattern
PFS: Progression-free survival
PRR: Pattern recognition receptor
s-OTU: Sub-operational taxonomic unit
