# Supplementary material for: Relating the gut metagenome and metatranscriptome to immunotherapy responses in melanoma patients
Source: Genome Med. 2019 Oct 9;11:61. doi: 10.1186/s13073-019-0672-4 (PMC6785875; doi:10.1186/s13073-019-0672-4)

**Additional file 2**

**Relating the gut metagenome and metatranscriptome to immunotherapy responses in melanoma patients**

Brandilyn A. Peters, Melissa Wilson, Una Moran, Anna Pavlick, Allison Izsak, Todd Wechter, Jeffrey S. Weber, Iman Osman, Jiyoung Ahn

^a^Department of Population Health, NYU School of Medicine, New York, NY, USA

^b^Department of Medicine, NYU School of Medicine, New York, NY, USA

^c^NYU Perlmutter Cancer Center, New York, NY, USA

^d^The Ronald O. Perelman Department of Dermatology, NYU School of Medicine, New York, NY, USA

**Figure S1. Patient clusters based on overall microbiome composition in 16S and shotgun data are related to progression-free survival.** Ward’s Hierarchical Agglomerative Clustering method was used on the Jensen-Shannon Divergence (JSD) from the 16S s-OTU data (a) and shotgun subspecies data (d) to cluster patients into groups. Principal coordinate analysis of the JSD showed separation of these patient groups for both the 16S (b) and shotgun (e) data. The Kaplan-Meier curves of the patient JSD clusters from both 16S data (c) and shotgun data (f) had significantly different progression-free survival (log-rank p = 0.005 for 16S JSD cluster, p = 0.02 for shotgun JSD cluster).


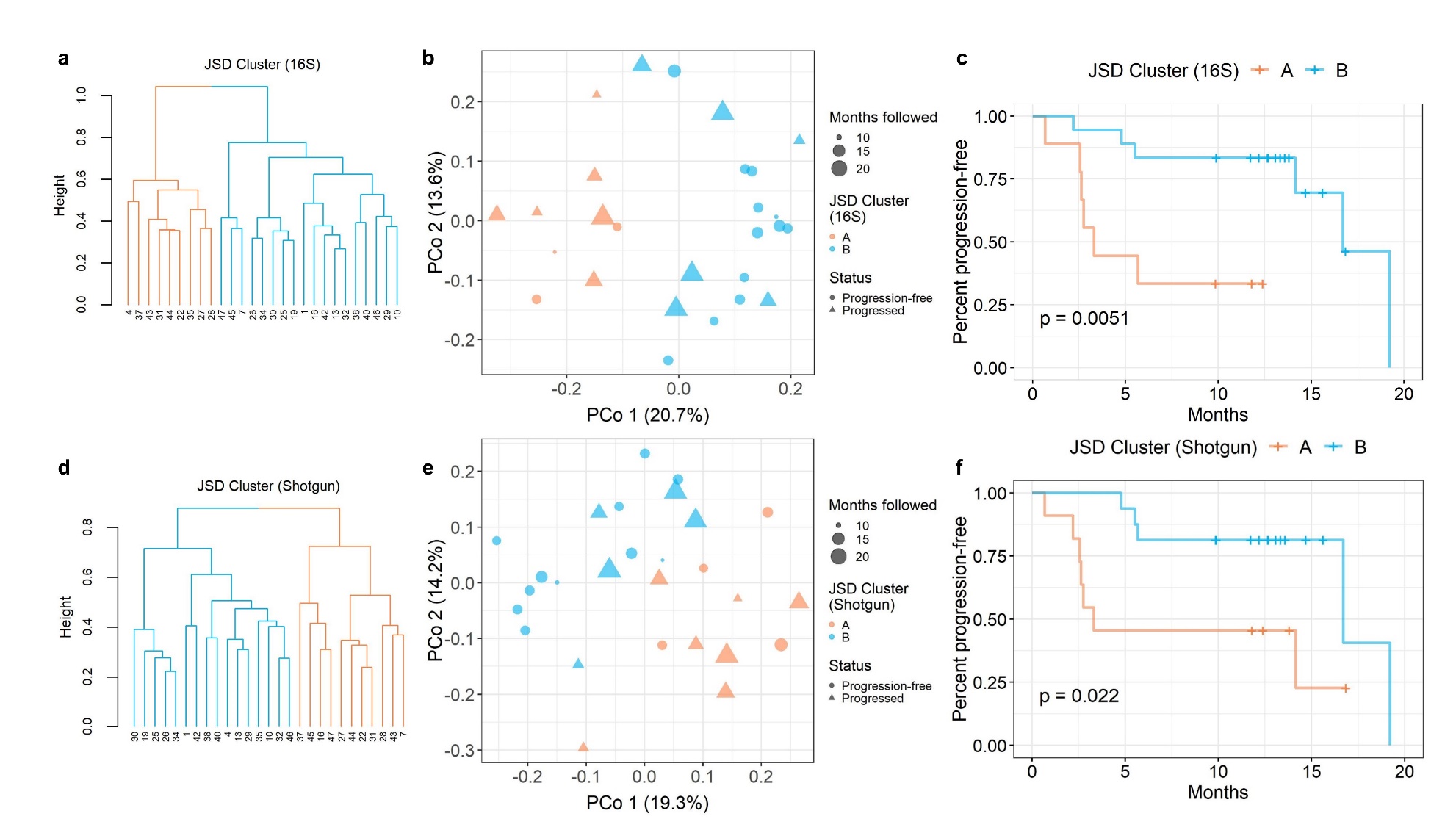


**Figure S2.** **Subspecies related to progression-free survival.** For shotgun subspecies selected >125 times in 500 x 10-fold cross-validated elastic-net penalized Cox regression and with FDR-adjusted q<0.20, we show number of times selected and the hazard ratio.


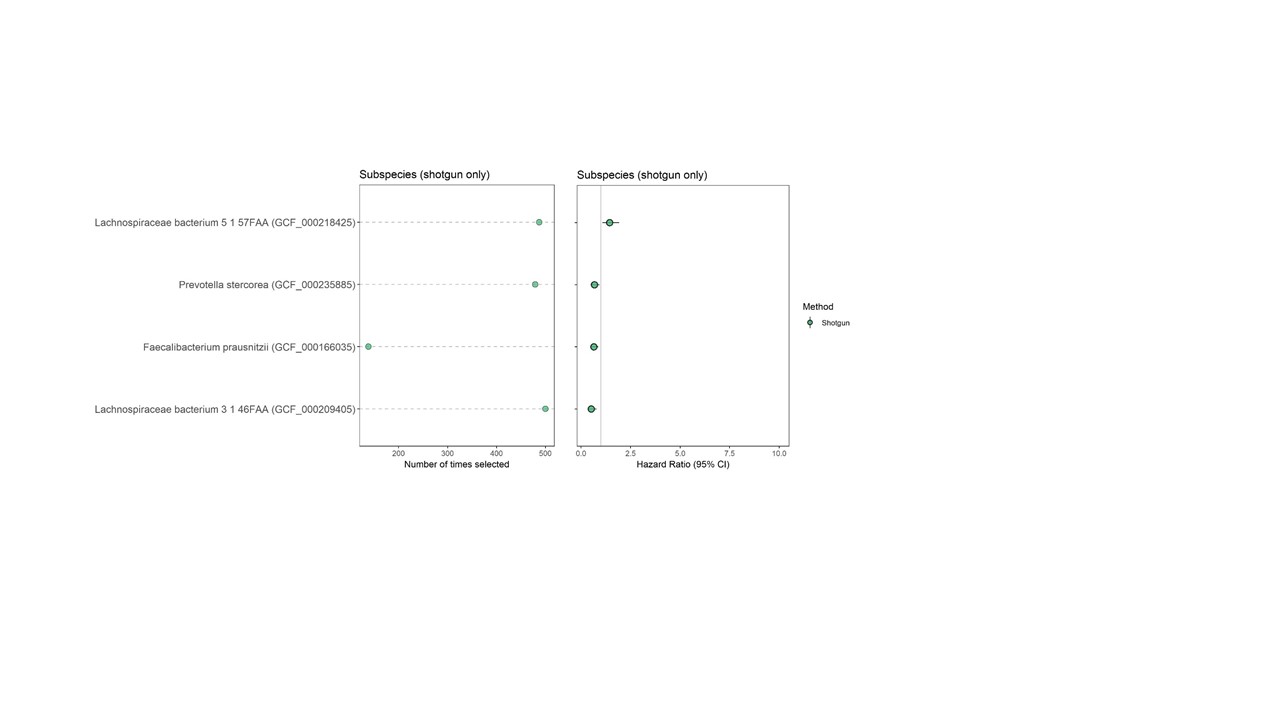


**Figure S3.** **Metagenomic reactions related to progression-free survival.** For metagenomic reactions selected >125 times in 500 x 10-fold cross-validated elastic-net penalized Cox regression, that also had FDR-adjusted q<0.20 and correlated (p<0.05) metatranscriptomic expression, we show (a) number of times selected and the hazard ratio (alongside parallel data from the metatranscriptomic analysis in n=17) and (b) correlations between metagenomic and metratranscriptomic reactions relative abundance. Spearman’s rho and p-value are displayed on the plots.


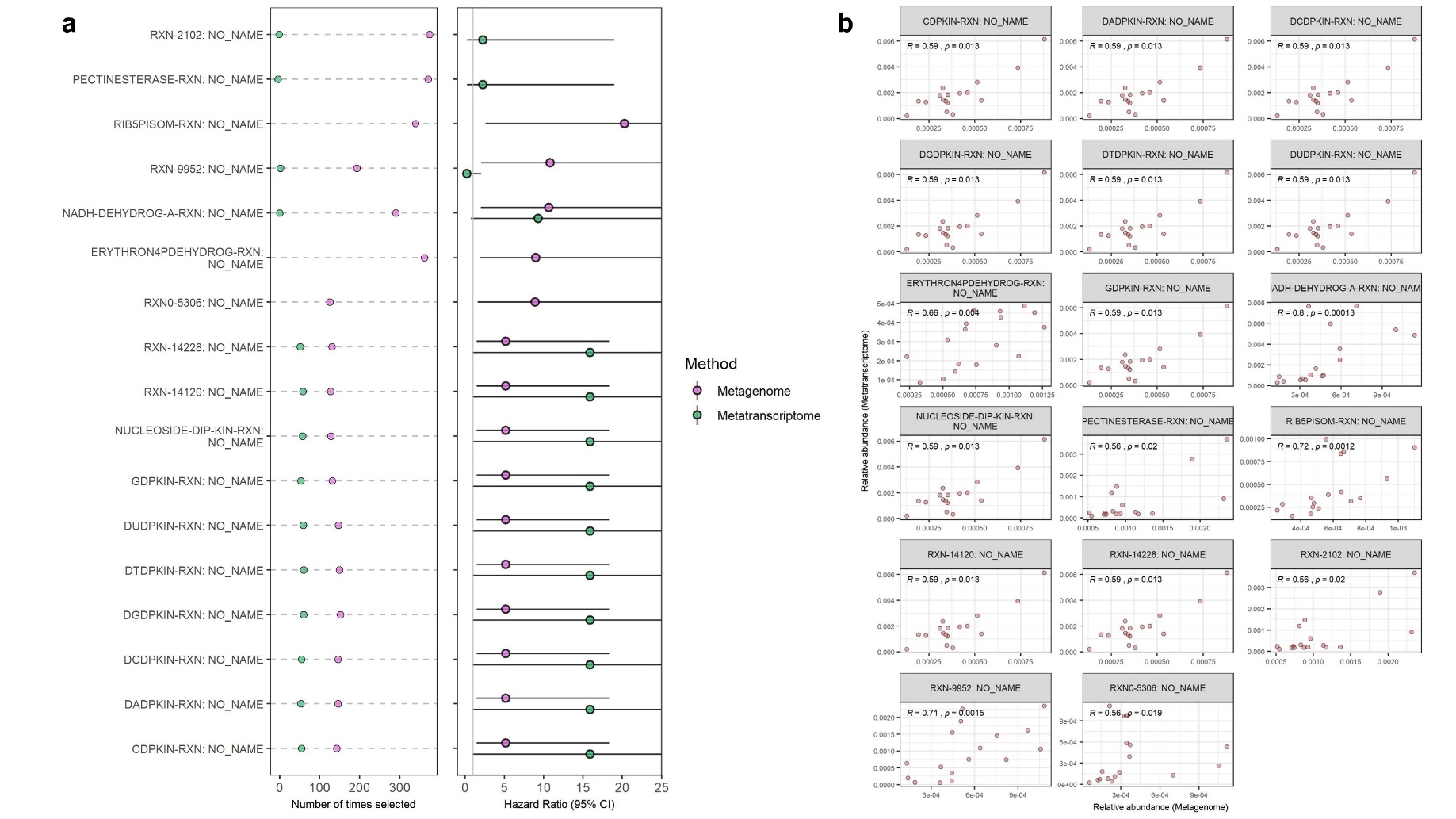


**Figure S4.** **Metagenomic gene families related to progression-free survival.** For metagenomic gene families selected >125 times in 500 x 10-fold cross-validated elastic-net penalized Cox regression, that also had FDR-adjusted q<0.20 and correlated (p<0.05) metatranscriptomic expression, we show (a) number of times selected and the hazard ratio (alongside parallel data from the metatranscriptomic analysis in n=17) and (b) correlations between metagenomic and metratranscriptomic gene families relative abundance. Spearman’s rho and p-value are displayed on the plots. **
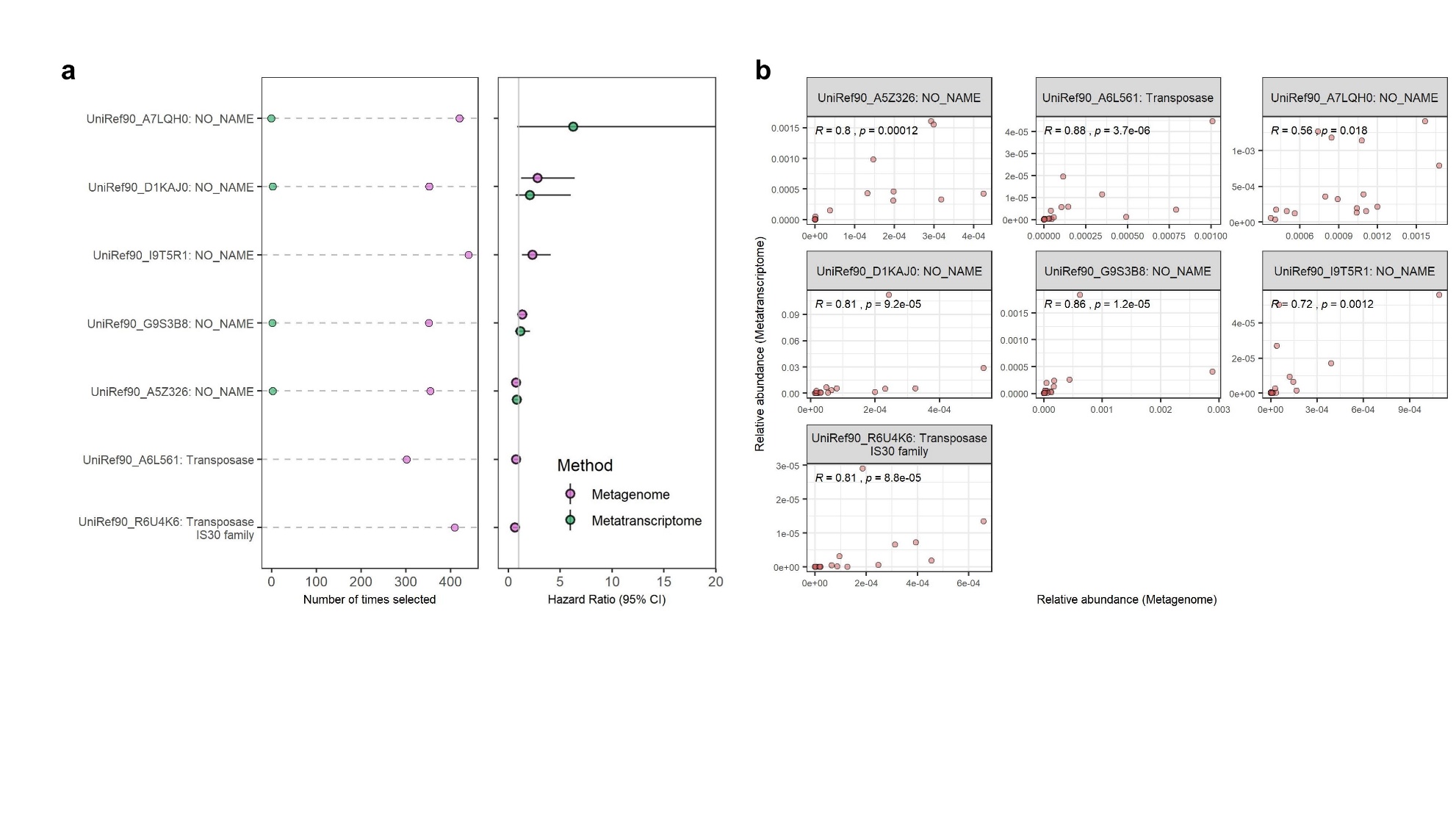
**

**Figure S5. Contribution of shotgun metagenome taxa to shotgun metagenome reactions.** (a) Spearman’s correlations are shown for shotgun species and subspecies vs. shotgun metagenome reactions and shotgun metatranscriptome reactions. Only taxa selected in repeated cross-validated elastic-net penalized Cox regression are shown, and only reactions selected in regression and that had correlated metatranscriptomic expression are shown. Taxa and reactions relative abundance were used for correlation analysis. Taxa and reactions are annotated with the direction of their hazard ratio with progression-free survival in the metagenome analysis. *p<0.05; **p<0.01. (b) Mean percent contribution of species to reactions in the metagenome and metatranscriptome data. Per species reaction abundance values were normalized to 100% for each reaction within each patient individually, and means were taken across patients; here we show the mean percent contribution for the top 5 contributing species to each reaction.

**
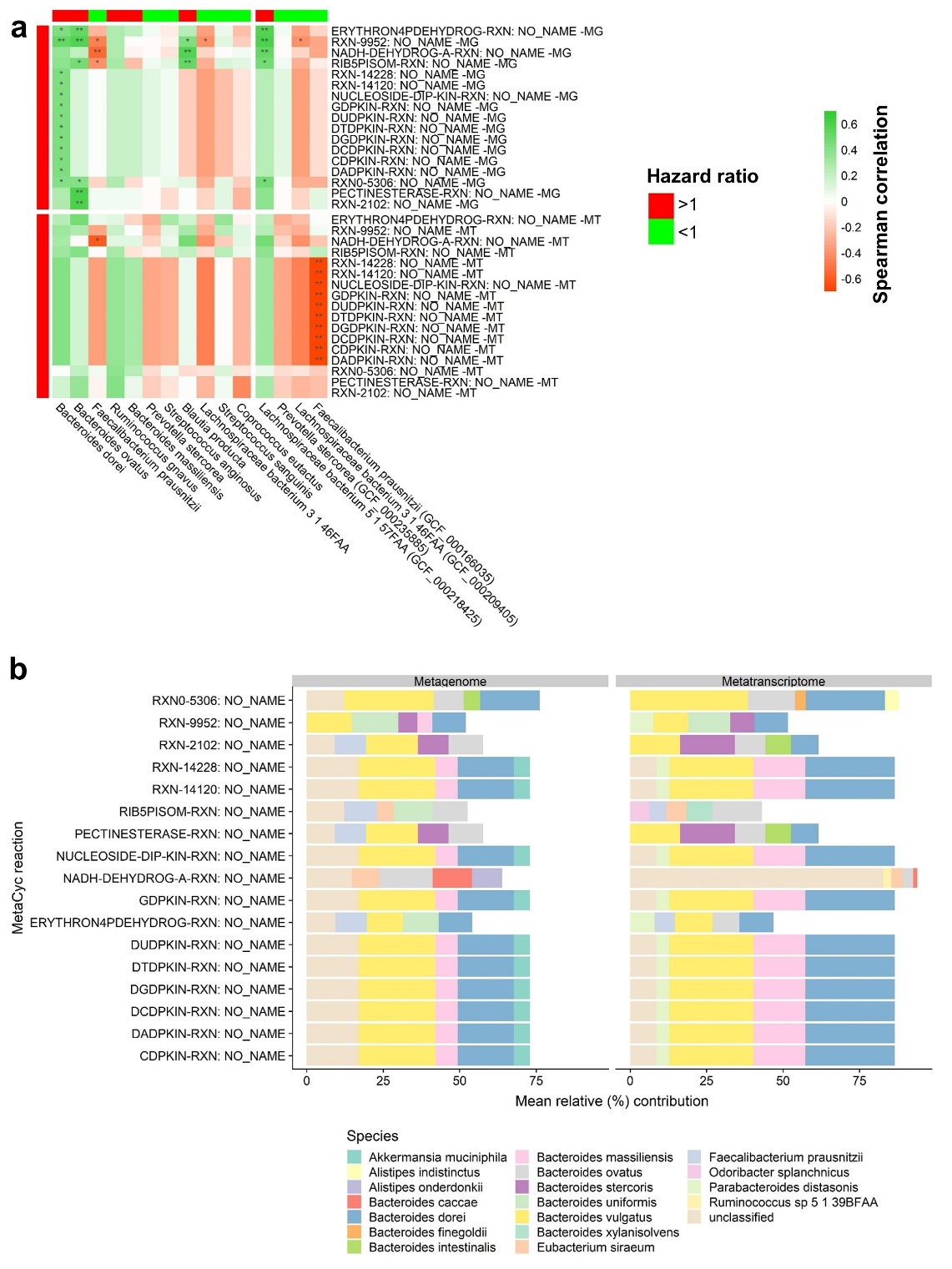
**

**Figure S6.** **Principal coordinate analysis of the Jensen Shannon Divergence.** Patient samples are colored based on adjuvant or metastatic status, with no significant difference between the two groups (PERMANOVA p = 0.16 adjusting for age, sex, BMI, stage, and antibiotics in last 6 months).


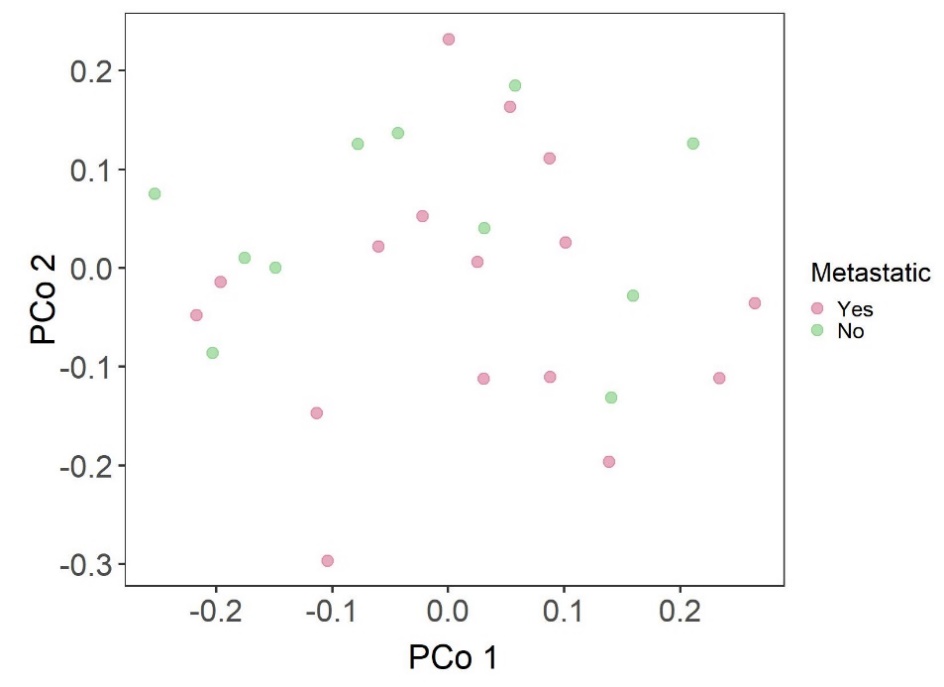

Supplement: Supplementary file 2 — Additional file 2. Supplementary Figures S1-S6. [file 13073_2019_672_MOESM2_ESM.docx]
